# Development of a novel Electrical Industry Safety Risk Index (EISRI) in the electricity power distribution industry based on fuzzy analytic hierarchy process (FAHP)

**DOI:** 10.1016/j.heliyon.2023.e13155

**Published:** 2023-01-30

**Authors:** Mohsen Sadeghi-Yarandi, Salman Torabi-Gudarzi, Nasrin Asadi, Hamedeh Golmohammadpour, Vahid Ahmadi-Moshiran, Mostafa Taheri, Aysa Ghasemi-Koozekonan, Ahmad Soltanzadeh, Bahare Alimohammadi

**Affiliations:** a Department of Occupational Health, School of Public Health, Tehran University of Medical Sciences, Tehran, Iran; b Department of Occupational Health Engineering, Behbahan Faculty of Medical Sciences, Behbahan, Iran; c The State University of New York, University of Buffalo, Department of Industrial Engineering, New York, USA; d Department of Occupational Health, School of Public Health, Hamadan University of Medical Sciences, Hamadan, Iran; e Islamic Azad University, Science and Research Branch, Department of Art and Architectural Engineering, Iran; f Department of Occupational Health, School of Public Health and Safety, Shahid Beheshti University of Medical Sciences, Tehran, Iran; g Department of Occupational Health, School of Public Health, Qom University of Medical Sciences, Qom, Iran; h Department of Ergonomics, University of Social Welfare and Rehabilitation Sciences, Tehran, Iran

**Keywords:** Risk assessment, Safety, Power distribution, Risk criteria, EISRI

## Abstract

Many workers are exposed to electrical energy during the fulfillment of their tasks. It is necessary to identify the potential risk factors for electrical damages. The present study aimed to develop a novel Electrical Industry Safety Risk Index (EISRI) in the electricity power distribution industry based on fuzzy analytic hierarchy process (FAHP). In this study several different safety risk assessment methods were analyzed. Then, common activities in the electricity distribution industry were classified into ten occupational groups. To identify the general structure of risk assessment and determine three main components, including personal, environmental, and organizational a three-stage Delphi study was conducted with the participation of 30 experts. The fuzzy analytic hierarchy process approach was used to weight the components and parameters in each job group. Finally, the results of the EISRI were compared with the failure mode and effect analysis (FMEA) method. The most effective component in determining the risk level was the personal component (PC), with a 0.537 weighted average. Cronbach's alpha values for each of the personal, environmental, and organizational components and the entire model were 0.90, 0.85, 0.82, and 0.86, respectively, and model reliability was confirmed. The results obtained from the EISRI method were compared with the FMEA method, the results of both methods were very close to each other (p < 0.05). The results of this study revealed that the highest weighted average was related to the personal component due to the high impact of the human factors in carrying out activities in various occupations. The EISRI can be applied as a substitute for general risk assessment methods due to the suitability of this method with the nature of activities in this industry. The present technique can be a practical step toward developing suitable risk management algorithm.

## 1 Introduction

Electricity is one of the fundamental demands of humans in both domestic and industrial fields and has always imposed many safety risks on individuals. Electricity technology and its development have induced a wide range of contact with electricity dangers for people, including technologists, workers involved in the production, transmission, and distribution of electricity, and ultimately consumers, including domestic and industrial customers [1,2]. In electricity power production, transmission, and distribution industries, electrical current exposes employees to severe occupational hazards and safety risk factors; practicaly all members of the labor force are exposed to electrical energy during their work shift. Among the different industries of electricity production, distribution and consumption, the electricity distribution industry is prone to more occupational safety risks due to the direct exposure of employees to power lines and electricity sources. Various incidents and accidents occur in the distribution sector of the electricity industry and cause numerous human and financial losses every year [[3], [4], [5]].

Electrocutions are among the most widespread injuries in industries. According to epidemiological research, 67% of all electrocutions are work-related. National Traumatic Occupational Fatality Database indicated that approximately 7% of the 6500 work-related traumatic fatalities that occur annually are duo to electrocutions [4]. According to the Occupational Safety and Health Administration (OSHA), 86% of losses in the production, transmission, and distribution industries are due to the effects of electricity, which leads to an average loss of 12,976 working days per year [6]. The highest death rate due to electricity occurs among the labor force in the electricity industry, especially electrical lineman [7].

The three main factors influencing electrical accidents are unsafe equipment, unsafe working environment, and unsafe operational procedure based on OSHA scenario analysis [8]. The National Institute for Occupational Safety and Health (NIOSH) studied 244 large accidents and presented five case scenarios that characterize electrical accidents: direct contact of employees with power lines (28%), direct contact of the employees with the electrical network equipment (21%), lift boom contact with power grid (18%), worn or damaged equipment of the power network (17%), and contact of conductive equipment with power lines (16%) [9].

Many workers are exposed to electrical energy during the performance of their tasks and sub-tasks. It is necessary to recognize the potential risk factors for electrical damages and provide useful suggestions for implementing effective safety programs to reduce the risk of electrocution.

A crucial part of any health and safety management system is identifying and assessing the various risks related to different hazard sources [7,10,11]. Risk assessment is a rational way of hazard evaluation that identifies risks and related potential consequences on individuals, materials, apparatus, and the environment [12]. Risk assessment methodologies provides worth data for decision-making process to reduce risk, determine acceptable risk levels, and create inspection and maintenance policies and standard operational procedures at industrial facilities [[13], [14], [15]].

There are several methods of risk identification and risk assessment [8,15,16]. Risk assessment methodologies are divided into two main category including quantitative and qualitative tools according to the applied parameters [7]. Quantitative methodologies aims to specify the degree of risk according to solely numerical or statistical values. In contrast, qualitative methodologies utilize parameters to assess the level of risk based on observations, categorial assessments, or non-numerical value [9]. Most available risk assessment tools calculates risks using two parameters of severity and probability [17]. Risk assessment tools include safety checklists, Job Safety Analysis (JSA), Hazard and Operability study (HAZOP), Failure mode and effects analysis (FMEA), Fault Tree Analysis (FTA), and many other methods that increase in number over time [18]. It is necessary to select a proper risk assessment methodology according to the situations in the industry and nature of working activities to prevent accidents effectively [19]. Due to the complex nature of accidents in different sectors, many countries are developing risk assessment techniques and their localization, for more accuracy and compatibility of the methods with the industrial process and nature of various occupational tasks and sub-tasks [20].

Several tools and techniques have been applied to identify and evaluate the risks of the electricity industry that have not been developed specifically for the electricity industry Studies have illustrated that research on safety risk assessment in electricity distribution and transmission was commonly performed based on human error and unsafe actions [22].

One of the most important ways to reduce various occupational incidents and accidents in electricity power distribution industries is to use risk management and assessment appropriate to the type of working situations. For this goal, in the first phase, all present possible hazards are identified, assesed, and evaluated, and eventually suitable control measures are taken according to the risk assessment output. Despite many researches were performed in the field of safety assessment in electricity power distribution industries, most of the available tools have particular complexities and goals. Occupational safety risk assessments to improve safety levels in these industries should be feasible and easy-to-use.

Morover, due to the nature of risk assessment process and the type of incidents that occur in electricity power distribution industries, there is no clear boundary and range to define the probability and severity components of these events, so appliying the proper tool that can specifically cover these risk levels is very crucial, and it will be helpful.

Therefore, due to the absence of a relatively comprehensive tool that can examine the most critical components and parameters affecting the probability and severity of accidents in the electricity power distribution industries as one of the most vulnerable and high-risk industries in the world, and also a need for a comprehensive and practical technique, the present study aimed to develop a novel Electrical Industry Safety Risk Index (EISRI) in the electricity power distribution industry based on fuzzy analytic hierarchy process (FAHP).

## 2 Method

This cross-sectional and practical study was conducted in 2022 using a semi-quantitative safety risk assessment technique in Gilan Power Distribution Company based on the fuzzy analytic hierarchy process (FAHP). This study was conducted according to the following steps.

### 2.1 The first phase

In this phase, FMEA (failure mode and effect analysis) and JSA (job safety analysis) methods were used to identify and primary assessment of the risks of the electricity distribution industry. A panel of experts grouped the jobs to determine the risks of different task and sub-tasks in the electricity distribution industry and, subsequently, more accurate risk assessment.

Before conducting the study, an informed consent form was obtained from the participants to participate in the study. Participants were able to leave the study at any stage of the research if they were not satisfied. In this study, no intervention or study was done on human or animal samples. This study were conducted according to established ethical guidelines of the electricity distribution company of Gilan province.

#### 2.1.1 Delphi study

To prepare the list of measured parameters according to the industry characteristics the Delphi method was utilized.

The Delphi method is a type of regular communication technique developed at first for forecasting according to the experts opinions. This methodology is a process for collecting the knowledge and wisdom existing to a group of experts, which is performed by distributing questionnaires among these population and giving response on the answers received. Participants in the Delphi study included 5 to 20 experts [15].

In the present study, a Delphi questionnaire was designed after determining the main parameters affecting the risk index. To capture the majority of the country's specialists cooperation, the opinions of 30 experts, including Ph.D. and MS graduates in the fields of occupational safety and health engineering, industrial management, and electrical engineering, employed in 25 universities and 15 large power plants and manufacturing industries, in three phase of Delphi study were gathered.

In the first phase of the Delphi study, experts were requested to comment on the method's overall structure. In the second phase, experts were requested to ranking components and parameters according to their importance. Then, to determine the importance of each of the criteria and sub-criteria in each of the job subgroups and to select the weight of each parameter, the analytic hierarchy process (AHP) was applied.

#### 2.1.2 Content validity

Content validity ratios (CVR) and content validity index (CVI) were used to evaluate the content validity quantitatively. In this regard, experts were asked to review each item based on a three-part range of “necessary,” “useful but not necessary,” and “not necessary” to determine CVR. The responses were calculated according to Eq. (1) [23].

(1) $$CVR=\frac{n_{E}-\frac{N}{2}}{\frac{N}{2}}$$

where, nE is the number of specialists who have answered the “necessary” option, and N is the total number of specialists. The CVI index was also calculated by Eq. (2) [24].

(2) $$CVI=\frac{\sum_{n}^{1}CVR}{R}$$

Where, $\sum_{n}^{1}CVR$ is the sum of the approving points for each item that scored " relevant but need review” and “completely relevant,” and R is the total number of specialists. After obtaining the tables from the experts, the CVR and CVI were calculated for each item and the items that did not get the minimum required score, were removed from the tables.

Content validity ratio (CVR) and content validity index (CVI) were used to determine the content validity of the model. The acceptable limit value of the CVR was considered 0.33 according to the Lawshe table and proportional to the number of participants in the Delphi study (30 experts) As shown in Table 1, the minimum validity score was calculated according to the number of expert panel [25]. Also, the acceptable limit value of the CVI was considered to be 0.79 [26].

**Table 1:** Minimum validity score according to the number of expert panel.

Number of Expert Panel	Minimum Validity Score
5	0.99
6	0.99
7	0.99
8	0.85
9	0.78
10	0.62
15	0.49
30	0.33

#### 2.1.3 Reliability

Cronbach's alpha method was used to evaluate the reliability of the new method [27]. An alpha coefficient of 0.7 or higher was considered as the minimum score required to confirm the model's reliability.

In this stage of the study, after determination of the general structure of risk assessment, the most effective causes of accidents were classified into three groups, including personal component, environmental component, and organizational component according to the results of previous studies and investigation of the accident database of electricity distribution industry in the last ten years (from 2012 to 2022).

At the end of this phase, after holding two sessions with panel members and using the information extracted from previous studies, the jobs in electricity distribution companies were divided into ten groups according to the nature of the job, stages of work, and similarity of risks, as described in Table 2. After job grouping, the hierarchical task analysis (HTA) and tabular task analysis (TTA) methods was used for the job analysis [20,22]. In order to use the participatory approach and also to identify all existing tasks and sub-tasks, after three training course, tabular task analysis (TTA) was also completed by the workers of the studied industry.

**Table 2:** Grouping of main job activities (Tasks) related to each job.

Row	Job	Main job activities
**1**	Electrician	- Medium air pressure network repairs (Cold Line) - Low air pressure network repairs (Cold Line) - Medium air pressure network repairs (Hot Line) - Low air pressure network repairs (Hot Line) - Ground medium pressure network repairs - Ground low pressure network repairs - Lighting of passages - Incidents repairs - Construction of air network - Construction of land network - Construction of air post - Construction of land post - Receipt of receivables (test, reading, cutting of branches, reading of heavy expenses) - Branching (hotline) - Branching (cold line)
**2**	Technical expert	- Visiting the installation site of measuring equipment - Test the two-way flow switches in the office unit - Thermography - Inspection of the network - Administrative affairs - Setting relays and types of power switches in the office unit - Amperage measurement
**3**	Office expert	- Administrative Affairs - Interacting with the client
**4**	Technician	- Periodic repair and service of transformers - Repair of all types of semi oil disconnections - Testing of all kinds of measuring equipment
**5**	Dispatching technician	- Leading executive groups - Working with computers
**6**	Driver	- Driving a car or vehicles - Loading goods and equipment
**7**	Services	- Serving tea - Cleaning the rooms of the building
**8**	Printing and copying	- Copying of papers
**9**	Security	- Monitoring and recording entrances and exits inside the office building
**10**	Warehouse	- Transportation and storage of various goods

### 2.2 The second phase

#### 2.2.1 Analytic hierarchy process (AHP)

Thomas L. Saaty presented this methodology in 1983. The aim of this method is to prioritize multiple criteria. The Analytic Hierarchy Process is a multi-criteria decision-making tool for weighting the parameters and choosing the optimal option. These criteria are paired based on study goals, and related weight is specified. Ultimately, the options are paired comparisons based on each criterion [28]. The main purpose of the hierarchical analysis process method is to opt the best option based on various criteria by developing a pairwise comparison matrix [29].

In the hierarchical process, the parameters of any level are compared in pairs with their respective parameters, and related weights are calculated (relative weights). In the Next phase, the final weight of each parameters is determined, which is called the absolute weight. In these comparisons, decision-makers will applied verbal judgments [15]. The comparison and weighing of factors are performed in a K × K matrix (K = number of rows and columns of the pairwise comparisons matrix). The pairwise comparison is performed according to the valuation of the row factor relevant to the column element. For valuation, an interval scale from 1 to 9 is usually utilize based on language phrases: a higher value shows the preference of a row element over a column element so that a value of 9 means the most valuable element, and a value of 1 means the least valuable element.

To enhancement the reliability of the findings of the questionnaires analysis, the consistent rate of the system is controlled. The reliability values of the expert panel questionnaire were considered the same as the adjustment rate. In this study, a value of 0.1 or less was considered as the acceptable compatibility limit of pairwise comparisons.

#### 2.2.2 Fuzzy logic

Fuzzy logic is a type of the multi-valued area in which the creteria's accurate values may be any real number between zero and one. This logic is use to apply the concept of partial correctness so that its values can be between fully accurate and fully false [30]. This method is a strong tool to deal with the uncertainty of human judgment in decision-making.

Different studies have used combining the AHP method with fuzzy logic to weight the criteria and sub-criteria. Various tools for performing FAHP have been suggested. This study was based on the method suggested by Chang because it is more comfortable to coduct than other approaches and supplies accurate outputs [31].

The Likert scoring method was used for the users scoring the sub-criteria during the activity (Table 3). With this range or scale, the respondents determine their level of agreement with the item. These levels are sequential and show the lowest to the highest agreement levels.

**Table 3:** Calculation of the Likert spectrum to assign a risk rating in **EISRI**.

Equivalent Likert spectrum			
**Totally inconsistent**	Often inconsistent	Often inconsistent	Totally consistent
**25%**	50%	75%	100%
Numerical equivalent			
**1**	2	3	4

#### 2.2.3 Formation of the risk matrix

The next step is to form a risk level matrix using the results obtained from the previous steps for each occupational group in the power distribution industry. The risk matrix is obtained by combining different levels of personal component, environmental component, and organizational component of varying levels of risk.

The risk matrix was divided into three levels by the ALARP (as low as reasonably practicable) principle and main criteria, including acceptable risks, tolerable risks, and unacceptable risks. ALARP” is short for “as low as reasonably practicable”. Reasonably practicable involves weighing a risk against the trouble, time, and money needed to control it. Thus, ALARP describes the level to which we expect to see workplace risks controlled. The ALARP concept arises within a regulatory framework. Increasingly, it is used by companies worldwide as it provides a reasonable basis for managing risks [32].

For this purpose, instead of forming a general risk matrix for all occupations, a separate risk matrix was drawn in each occupational group and used to determine the three levels of the risks according to experts' opinions. After receiving the final information, ten risk matrices with three levels were designed for occupational groups.

### 2.3 The third phase

In this phase of the study, in each job group, two most frequent tasks were evaluated using both the FMEA and the new method in Gilan Power Distribution Company to assess the efficiency and validity of the new method by caparison of the results.

Some occupational group activities such as the electrician with climbing height task, a technical with a panel-loading task, service personnel with the general cleaning task, the administrative expert during work on computer, and driving were selected for risk assessment using the FMEA method. Each activity was observed and evaluated in three different periods, then the average of the results of three observations was considered as the final score. After determining the hazards, the possible consequences and their root causes were identified. Then, the risk priority number (RPN) was determined based on the intensity, repeatability, and risk detection capability.

Finally, in order to provide descriptive indicators, average, standard deviation and frequency statistics were used. In order to check the relationship between the risk levels of the new method and the FMEA method, chi-square test was used at a significance level of 0.05. All analyzes were performed in the environment of SPSS software version 25.

## 3 Results

A total of 30 experts were involved in the present study. The expert panel's mean age and work experience were 41.63 ± 8.09 and 8.12 ± 5.13 years, respectively. 51% of the experts were Ph.D., and 49% had an MS degree.

As shown in Table 4, by conducting an initial risk assessment, the most important causes of accidents were identified and categorized into three groups: personal, environmental, and organizational components.

**Table 4:** Final component (criteria) and parameters (sub-criteria) in **EISRI**.

The main components (Criteria)	Parameters (Sub-Criteria)
Personal component	1. The work is not done individually
	2. There is no haste during the work shift
	3. There is the necessary skill and knowledge to perform the activities
	4. Personal and group protection devices are used
	5. There is physical competence to do the job
Environmental component	1. There are no hazardous work situations (harmful factors)
	2. The equipment and facilities are not old and worn out
	3. Personal and group protection equipment are available
	4. The work atmosphere is favorable
	5. There are no complicated and unfamiliar equipment and working conditions
Organizational component	1. There is no excessive time pressure in performing tasks
	2. The required equipment, facilities, and knowledge are exist
	3. There are risk identification and risk assessment programs
	4. Design, planning, organized responsibilities and supervision exist
	5. Lack of concern for safety and the tendency of supervisors and the organization to time and economic issues
	6. Monitoring, inspection, and auditing exist

The results of CVR and CVI for parameters affecting personal, environmental, and organizational components are presented in Table 5. In this table, 16 effective factors are listed that have an acceptable result for their content validity (see Table 6).

**Table 5:** Content validity review (CVR, CVI).

Factors affecting personal, environmental, and management components	CVR	Result	CVI
1. Haste and acceleration while working	0.99	A	0.99
2. Doing work individually	0.99	A	0.87
3. Lack or deficiency of skills and knowledge to perform activities	0.75	A	0.99
4. Not using the personal and group protection equipment	0.99	A	0.99
5. Lack of physical and mental competence to do work	0.99	A	0.87
6. Old and worn equipment and facilities	0.99	A	0.99
7. Dangerous work situations (ergonomic, physical (such as heat and cold) and mechanical hazards)	0.75	A	0.99
8. Complex and unfamiliar equipment and working conditions	0.80	A	0.80
9. Lack or incompleteness of personal or group protective equipment.	0.99	A	0.87
10. Unfavorable workplace atmosphere	0.99	A	0.99
11. Lack or non-compliance of existing equipment, facilities, and knowledge with existing demands	0.75	A	0.99
12. Excessive pressure on the performance, high and illogical personal workload	0.75	A	0.87
13. Incomplete or lack of risk identification and risk assessment program related to technical activities	0.75	A	0.87
14. Lack or incompleteness of design and planning, organization, responsibilities and supervision and distribution of work among individuals and failure to do work systematically	0.75	A	0.87
15. Lack of concern for safety and the tendency of supervisors and the organization to time and economic issues,lack of attention to safety aspects	0.80	A	0.80
16. Lack of supervision, inspections, and audits	0.80	A	0.87

*A: Acceptable.

**Table 6:** Final weight of sub-criteria affecting the **EISRI**.

Job	Main Component	Weight of Main Component	Main Parameters					
Electrician	a WPC	0.70	d P1	P2	P3	P4		P5
			0.09	0.27	0.06	0.20		0.08
	b WEC	0.13	e E1	E2	E3	E4		E5
			0.03	0.01	0.04	0.03		0.02
	c WOC	0.17	f O1	O2	O3	O4	O5	O6
			0.03	0.04	0.02	0.03	0.04	0.01
Technical expert	WPC	0.51	P1	P2	P3	P4		P5
			0.06	0.12	0.07	0.18		0.08
	WEC	0.29	E1	E2	E3	E4		E5
			0.05	0.06	0.04	0.09		0.05
	WOC	0.20	O1	O2	O3	O4	O5	O6
			0.04	0.04	0.02	0.03	0.04	0.03
Office expert	WPC	0.49	P1	P2	P3	P4		P5
			0.04	0.10	0.13	0.08		0.14
	WEC	0.20	E1	E2	E3	E4		E5
			0.04	0.03	0.04	0.05		0.04
	WOC	0.31	O1	O2	O3	O4	O5	O6
			0.07	0.05	0.06	0.05	0.05	0.03
Technician	WPC	0.53	P1	P2	P3	P4		P5
			0.10	0.12	0.06	0.21		0.04
	WEC	0.25	E1	E2	E3	E4		E5
			0.06	0.05	0.04	0.06		0.04
	WOC	0.22	O1	O2	O3	O4	O5	O6
			0.06	0.04	0.02	0.03	0.03	0.04
Dispatching technician	WPC	0.47	P1	P2	P3	P4		P5
			0.04	0.15	0.08	0.06		0.14
	WEC	0.25	E1	E2	E3	E4		E5
			0.05	0.05	0.04	0.08		0.03
	WOC	0.28	O1	O2	O3	O4	O5	O6
			0.08	0.05	0.05	0.03	0.05	0.02
Driver	WPC	0.65	P1	P2	P3	P4		P5
			0.04	0.27	0.13	0.12		0.09
	WEC	0.25	E1	E2	E3	E4		E5
			0.07	0.04	0.03	0.07		0.04
	WOC	0.10	O1	O2	O3	O4	O5	O6
			0.02	0.02	0.02	0.01	0.01	0.02
Services	WPC	0.50	P1	P2	P3	P4		P5
			0.07	0.13	0.08	0.12		0.10
	WEC	0.36	E1	E2	E3	E4		E5
			0.09	0.09	0.06	0.08		0.04
	WOC	0.14	O1	O2	O3	O4	O5	O6
			0.05	0.03	0.01	0.02	0.02	0.01
Printing and copying	WPC	0.49	P1	P2	P3	P4		P5
			0.04	0.08	0.13	0.15		0.09
	WEC	0.40	E1	E2	E3	E4		E5
			0.12	0.08	0.09	0.07		0.04
	WOC	0.11	O1	O2	O3	O4	O5	O6
			0.03	0.01	0.02	0.02	0.01	0.02
Warehouse	WPC	0.51	P1	P2	P3	P4		P5
			0.03	0.18	0.08	0.17		0.05
	WEC	0.39	E1	E2	E3	E4		E5
			0.09	0.11	0.08	0.06		0.05
	WOC	0.10	O1	O2	O3	O4	O5	O6
			0.03	0.01	0.02	0.01	0.02	0.01
Security	WPC	0.52	P1	P2	P3	P4		P5
			0.15	0.11	0.09	0.09		0.08
	WEC	0.30	E1	E2	E3	E4		E5
			0.07	0.03	0.05	0.11		0.04
	WOC	0.18	O1	O2	O3	O4	O5	O6
			0.05	0.04	0.01	0.03	0.02	0.03

a Weight of the personal component.

b Weight of the environmental component.

c Weight of organizational component.

d Sub-criteria of personal component.

e Sub-criteria of environmental component.

f Sub-criteria of organizational component.

Cronbach's alpha values for each of the personal, environmental, and organizational components and the entire model were 0.90, 0.85, 0.82, and 0.86, respectively, and model reliability was confirmed.

### 3.1 Electical Industry Safety Risk Index (EISRI)

The numerical value of Electical Industry Safety Risk Index (EISRI) was estimated using Eq. (3):

(3) EISRI=100−((PC+EC+OC)×100)

where PC is the personal component, EC is the environment component, and OC is the organizational component. Equations No. 4, 5, and 6 were used to obtain each of three main components. To calculate the effective weight of the sub-criteria, the score of the main criterion was multiplied by the weight of the relevant sub-criterion. Then by summing the effective weight of the sub-criteria, the weight of the main criteria was calculated.

• P_1_ = W_P1_ × Score
• P_2_ = W_P2_ × Score
• P_3_ = W_P3_ × Score
• P_4_ = W_P4_ × Score
• P_5_ = W_P5_ × Score

(4) $$W_{PC}=P_{1}+P_{2}+P_{3}+P_{4}+P_{5}$$

• E_1_ = W_E1_ × Score
• E_2_ = W_E2_ × Score
• E_3_ = W_E3_ × Score
• E_4_ = W_E4_ × Score
• E_5_ = W_E5_ × Score

(5) $$W_{PC}=E_{1}+E_{2}+E_{3}+E_{4}+E_{5}$$

• O_1_ = W_O1_ × Score
• O_2_ = W_O2_ × Score
• O_3_ = W_O3_ × Score
• O_4_ = W_O4_ × Score
• O_5_ = W_O5_ × Score

(6) $$W_{MC}=M_{1}+M_{2}+M_{3}+M_{4}+M_{5}$$

where P is sub-criteria of personal component, E is sub-criteria of environmental component, M is sub-criteria of organizational component, W_PC_ is the weight of the personal component, W_EC_ is the weight of the environmental component, and W_OC_ is the weight of organizational component, and the score is the weight of the main criterion.

**Fig. 1:**
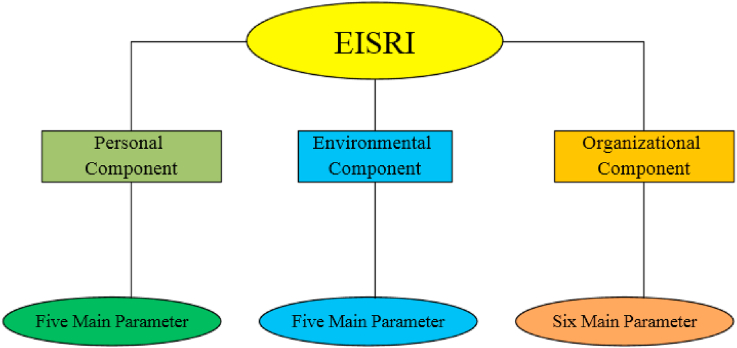
The schematic of the new risk assessment method.

The schematic of the new risk assessment method is shown in Fig. 1.

### 3.2 Risk matrix

The risk matrix was divided into three levels by the ALARP (as low as reasonably practicable) principle and main criteria, including acceptable risks, tolerable risks, and unacceptable risks. For maximum accuracy in estimating the risk level, instead of forming a general risk matrix for all occupations, a separate risk matrix was drawn in each occupational group. For instance, the electrician matrix is shown in Table 7, and the other matrix tables are in the appendix (see Tables S1–S10).

**Table 7:** Risk matrix for Electrician job in **EISRI**.

Electrician	EC				
**PC**	100	83.9	60	40	20
	96	76	56	36	16
	92	72	52	32	12
	88	68	48	28	8
	84	67.9	44	24	4
**OC**

**Table 8:** Comparison of the results of risk assessment with FMEA and EISRI.

Occupational group	Electrician	Technician	Office expert	Service	Driver	P-Value
Type of activity	Climb to height	Download from the board	Work with computer	General cleaning	Driving cars	
**Risk Number in EISRI**	23.42	50.31	10.20	24.16	20.13	0.001^a^
**Risk Priority Number in FMEA**	130	312	101	99	133	

a Chi-square (p < 0.05); Significant correlation.

### 3.3 Analytical results

The results revealed that the most important main component in determining the level of risk in all ten occupational groups was the PC, with an average weight of 0.537.

In personal component, the average weight of the most effective sub-criteria were according to following.

1 The weight of haste while working in the job groups of the electrician, technical expert, driver, dispatching technician, service, and warehouse were 0.27, 0.12, 0.27, 0.15, 0.13 and 0.18, respectively.
2 The weight of physical competence to work in the administrative expert group was 0.14
3 The weight of personal and group protective equipment in the occupational group of the technical technician was 0.21.
4 The weight of personal and group protective equipment in the occupational group of printing and copying was 0.15.
5 The weight of performing work individually in the security group was 0.15.

P, E, and M are the final weight of the sub-criterion (multiplied by the value of the sub-criterion in the main criterion in each job group).

The results obtained from the developed risk assessment method were compared with the FMEA method and presented, the results of both methods were very close to each other (p < 0.05).

## 4 Discussion

The electricity distribution industry is one of the most important industries in the world, and many accidents occur in this industry every year, which causes many human and financial damages to various organizations and societies. Therefore, formulation, development and application of a safety index evaluation method in different parts of this industry and according to the nature of activities in this industry, for the implementation of corrective and control measures, is of great importance.

In the present study, the jobs in electricity distribution companies were divided into ten groups according to the nature of the job and related activities. This study also considered several criteria in job grouping, including similarity of job activities, the risk of the jobs, the importance of jobs, degree of involvement in activities of the company, and the experts’ comments. Then, the tasks of each occupational group were categorized, and the risks and possible events were determined using the Hierarchical Task Analysis (HTA) and tabular task analysis (TTA) methods. The Delphi method and the fuzzy analytic hierarchy process (FAHP) approach were used to determine the criteria and sub-criteria affecting the level of risk and related weighting.

Previous studies have also illustrates that the use of the Delphi study approach and the fuzzy analytic hierarchy process (FAHP) method can be an effectual step in developing semi-quantitative and quantitative risk assessment methodologies based on the characteristics of different work environment situations [15]. A study performed by Pinto et al. showed that health and safety risk assessment is a complex task for employees and requires considering multiple and appropriate parameters for each job [33].

The findings of Nasrollah Nejhada et al. study in an electricity distribution company of Iran revealed that the majority of the accidents occurred in summer. Workers’ negligence was the cause of 75% of deaths. Also, their findings indicated that occupational injuries are going to have an increase in the future [34]. These findings show the need to use risk assessment methods appropriate to the nature of this industry.

The present study showed that the most influential criterion in the risk level is personal component (PC) in all ten occupational groups. The reason for this may be the nature of the job in electricity distribution companies and the important role of personal behaviors in the risk occurrence. By studying work-related accidents, it can be concluded that more than 90% of accidents are due to human behavior and error, which confirms the results of the present study [35].

The present study indicated that the most influential factors in the occurrence of accidents.

1. The personal component includes working individually, not using personal and group protection equipment, haste while doing work, and lack of necessary knowledge and skills to perform activities.
2. The environmental component includes dangerous work situations and harmful environmental factors, worn out and old equipment and facilities, unfavorable work atmosphere, and complex and unfamiliar equipment and facilities.
3. The organizational component includes excessive pressure on workers to perform their duties, lack of risk identification and effective risk assessment program, lack of supervision and organized tasks and responsibilities, lack of safety concerns, the tendency of managers to economic issues, and the lack of adequate and regular inspections and audits.

The results of this study are consistent with the results of some studies about the risks of low-voltage networks [35,36]. The results of Tiju Baby et al.'s study demonstrated that personal factors, safety climate factors, and health conditions of the workers were significantly associate to occupational electrical accidents. In personal component, age, job role, education, and experience are significantly effecting the safety behaviour of the electrical workers. The results of the Baby et al.'s study highlighted the need for interventions to reduce personal parametes in the electrical industry [5]. In the current study, the highest weighted average was related to the personal component, which can be an effective step in highlighting the role of this component in the risk score and finally implementing control measures.

The results revealed that the most important main component in determining the level of risk in all ten occupational groups was the PC, with an average weight of 0.537.

In the present study, after weighing the criteria and sub-criteria through pairwise comparisons, experts' opinions were quantitatively summed, and the data were normalized. The results showed that the weight of haste while working in the job groups of the electrician, technical expert, driver, dispatching technician, service, and warehouse were 0.27, 0.12, 0.27, 0.15, 0.13 and 0.18, respectively and has the most significant effect in the personal component. The reason for this issue can be considered as having high concentration as a job requirement in the mentioned jobs. When people are in a hurry, their attention is affected, significantly increasing the risk of hazards.

The sub-criterion of physical competence to work in the job group of the administrative employee with an average weight of 0.14 has the most significant impact on the risk level in this job group. Office employees spend most of their time sitting and working with a computer. Suppose the ergonomic principles are not observed or the work station is not appropriate, including the fit of the desk and chair with the person. In that case, it can lead to musculoskeletal disorders over time. One of the essential factors in creating a fit for the person in the work environment is being in the standard and ergonomic design range. If the ergonomic principles is not observed, the complications will appear over time. Previous studies conducted to determine the risk factors associated with the prevalence of musculoskeletal disorders, revealed that the incompatibility of the person and the work station leads to unfavorable ergonomic conditions and increased risk of ergonomic disorders [37,38]. Also, in a study conducted by Suri et al. to investigate the relationship between quality of life and the incidence of musculoskeletal disorders in car assemblers, it was found that the physical condition of individuals is effective in the occurrence of ergonomic risks [39].

The sub-criterion of personal and group protection equipment in the occupational group of technical technicians with an average weight of 0.21 was determined as the most effective sub-criterion in the risk occurrence. This job group has good knowledge and skills due to specialized job activities. On the other hand, the workload in this job group is usually pre-determined, and sub-criteria such as haste during work have negligible effects on the risk level. In this occupational group, personal and group protection equipment as the main barrier between risk and individuals is an essential factor in preventing accidents.

The sub-criterion of barriers and personal and group protection equipment in the printing and copying group with an average weight of 0.15 was identified as the most effective sub-criterion in risk occurrence. In this occupational group, exposure to harmful chemical factors such as gases and vapors is possible during paper copying. They should use personal protective equipment such as respirators, gloves, and general ventilation to prevent health risks. Performing work individually in the security group with an average weight of 0.15 was determined as the most effective sub-criterion in the risk of accidents.

Ultimately, the results of the new risk assessment method were similar to the results obtained from the FMEA, which shows the reliability of the results obtained for specialized risk assessment in the electricity industry.

### 4.1 Limitations and strengths of the study

Among the limitations of the current method is the lack of a quantitative approach to calculate and evaluate the effective parameters of the electricity distribution industry's probability and severity of risks. Thus, it is suggested that researchers in the future develop and apply quantitative tools according to the conditions of using safety management systems via international management guidelines.

Another limitations of this study was not considering some of the activities done in different ways in different electricity distribution companies in the country. It is necessary to continue this study with a full review of jobs in this industry and develop this risk assessment approach for all activities in electricity companies all over the world.

The present study was performed for the first time to introduce and implement a unique approach to assess electricity distribution industry safety risk and accordance with the particular specification of this industries and activities. Among the strengths of this study are the risk assessment at the place of activity and the presentation of controls immediately to take rapid measures to reduce the risk level. Also, control measures are presented separately in three personal, environmental, and organizational components, making the rules more effective and easier to implement.

Another Strength of the present study is forming a specific risk matrix for each job, considering the nature of the job and the importance and severity of accidents in different occupations, ultimately leading to a more accurate estimate of the risk level, which differs from many existing safety risk estimation methods.

This study determined the most important factors affecting occupational accident frequency and severity. The present technique can be a practical step toward decreasing occupational accident risk levels in the electricity distribution industry and developing control plans, especially in developing countries, due to low safety performance and related inspections.

## 5 Conclusion

The present method was developed under the title of “Electical Industry Safety Risk Index (EISRI)" based on three personal, environmental and organizational components and using 16 sub-criteria or parameters using the fuzzy analytic hierarchy process (FAHP) approach. The results of the present study showed that the highest weighted average was related to the personal component due to the high impact of the human factors in carrying out activities in various occupations. The EISRI can be used as a substitute for general risk assessment methods due to the suitability of this method with the nature of activities in this industry**.** The present technique can be a practical step toward decreasing occupational accident risk levels in the electricity distribution industry and developing control plans, especially in developing countries, due to lower risk management performance.

## Declarations

### Credit author statement

Mohsen Sadeghi-Yarandi: Conceived and designed the experiments; Wrote the paper.Salman Torabi-Gudarzi: Performed the experiments; Contributed reagents, materials, analysis tools or data; Wrote the paper.Nasrin Asadi, Hamedeh Golmohammadpour: Analyzed and interpreted the data.Vahid Ahmadi-Moshiran: Performed the experiments; Analyzed and interpreted the data.Mostafa Taheri, Aysa Ghasemi-Koozekonan: Performed the experiments.Ahmad Soltanzadeh: Contributed reagents, materials, analysis tools or data.Bahare Alimohammadi: Conceived and designed the experiments.

### Funding statement

This research did not receive any specific grant from funding agencies in the public, commercial, or not-for-profit sectors.

### Data availability statement

Data included in article/supp. Material/referenced in article.

## Declaration of competing interest

The authors declare that they have no known competing financial interests or personal relationships that could have appeared to influence the work reported in this paper.
