# Supplementary material for: Development of a novel Electrical Industry Safety Risk Index (EISRI) in the electricity power distribution industry based on fuzzy analytic hierarchy process (FAHP)
Source: Heliyon. 2023 Jan 30;9(2):e13155. doi: 10.1016/j.heliyon.2023.e13155 (PMC9900264; doi:10.1016/j.heliyon.2023.e13155)
Supplement: Multimedia component 1 [file mmc1.docx]

Table S1_,_ Risk matrix of Electrician

| ECL | | | | | **Electrician** |
| --- | --- | --- | --- | --- | --- |
|  |  |  |  |  |  |
| 20 | 40 | 60 | 80 | 100 | PCL |
| 16 | 36 | 56 | 76 | 96 |  |
| 15/9 | 32 | 52 | 72 | 92 |  |
| 8 | 31/9 | 48 | 68 | 88 |  |
| 4 | 24 | 44 | 64 | 84 |  |
| OCL | | | | | |
|  |  |  |  |  |  |

Table S2_,_ Risk matrix of Technical expert

| ECL | | | | | **Technical expert** |
| --- | --- | --- | --- | --- | --- |
|  |  |  |  |  |  |
| 20 | 40 | 60 | 80 | 100 | PCL |
| 16 | 36 | 56 | 76 | 96 |  |
| 12 | 32 | 52 | 72 | 92 |  |
| 8 | 28 | 48 | 71.9 | 88 |  |
| 4 | 27/9 | 47/9 | 64 | 84 |  |
| OCL | | | | | |
|  |  |  |  |  |  |

Table S3_,_ Risk matrix of Office expert

| ECL | | | | | Office expert |
| --- | --- | --- | --- | --- | --- |
|  |  |  |  |  |  |
| 20 | 40 | 60 | 80 | 100 | PCL |
| 16 | 36 | 56 | 76 | 96 |  |
| 12 | 32 | 55/9 | 72 | 92 |  |
| 8 | 28 | 48 | 71.9 | 88 |  |
| 4 | 27/9 | 44 | 64 | 84 |  |
| OCL | | | | | |
|  |  |  |  |  |  |

Table S4_,_ Risk matrix of Technician

| ECL | | | | | Technician |
| --- | --- | --- | --- | --- | --- |
|  |  |  |  |  |  |
| 20 | 40 | 60 | 80 | 100 | PCL |
| 19/9 | 36 | 56 | 76 | 96 |  |
| 12 | 35/9 | 52 | 72 | 92 |  |
| 8 | 28 | 48 | 68 | 88 |  |
| 4 | 24 | 44 | 64 | 84 |  |
| OCL | | | | | |
|  |  |  |  |  |  |

Table S5_,_ Risk matrix of dispatching technician

| ECL | | | | | Dispatching technician |
| --- | --- | --- | --- | --- | --- |
|  |  |  |  |  |  |
| 20 | 40 | 60 | 80 | 100 | PCL |
| 16 | 36 | 59/9 | 76 | 96 |  |
| 12 | 32 | 52 | 72 | 92 |  |
| 8 | 31/9 | 48 | 68 | 88 |  |
| 4 | 24 | 44 | 64 | 84 |  |
| OCL | | | | | |
|  |  |  |  |  |  |

Table S6_,_ Risk matrix of driver

| ECL | | | | | Driver |
| --- | --- | --- | --- | --- | --- |
|  |  |  |  |  |  |
| 20 | 40 | 60 | 80 | 100 | PCL |
| 16 | 39/9 | 56 | 76 | 96 |  |
| 15/9 | 32 | 52 | 72 | 92 |  |
| 8 | 28 | 48 | 68 | 88 |  |
| 4 | 24 | 44 | 64 | 84 |  |
| OCL | | | | | |
|  |  |  |  |  |  |

Table S7_,_ Risk matrix of Services

| ECL | | | | | Services |
| --- | --- | --- | --- | --- | --- |
|  |  |  |  |  |  |
| 20 | 40 | 60 | 80 | 100 | PCL |
| 19/9 | 36 | 56 | 76 | 96 |  |
| 12 | 32 | 52 | 75.9 | 92 |  |
| 8 | 28 | 48 | 68 | 88 |  |
| 4 | 24 | 47.9 | 64 | 84 |  |
| OCL | | | | | |
|  |  |  |  |  |  |

Table S8_,_ Risk matrix of Printing and duplication

| ECL | | | | | Printing and duplication |
| --- | --- | --- | --- | --- | --- |
|  |  |  |  |  |  |
| 20 | 40 | 60 | 80 | 100 | PCL |
| 16 | 36 | 56 | 76 | 96 |  |
| 12 | 32 | 55/9 | 72 | 92 |  |
| 8 | 31/9 | 48 | 68 | 88 |  |
| 4 | 24 | 44 | 64 | 84 |  |
| OCL | | | | | |
|  |  |  |  |  |  |

Table S9_,_ Risk matrix of warehouse

| ECL | | | | | warehouse |
| --- | --- | --- | --- | --- | --- |
|  |  |  |  |  |  |
| 20 | 40 | 60 | 80 | 100 | PCL |
| 19/9 | 36 | 56 | 76 | 96 |  |
| 12 | 32 | 52 | 72 | 92 |  |
| 8 | 28 | 48 | 68 | 88 |  |
| 4 | 24 | 47/9 | 64 | 84 |  |
| OCL | | | | | |
|  |  |  |  |  |  |

Table S10_,_ Risk matrix of Security

| ECL | | | | | Security |
| --- | --- | --- | --- | --- | --- |
|  |  |  |  |  |  |
| 20 | 40 | 60 | 80 | 100 | PCL |
| 16 | 36 | 59/9 | 76 | 96 |  |
| 12 | 32 | 52 | 72 | 92 |  |
| 8 | 31/9 | 48 | 68 | 88 |  |
| 4 | 24 | 44 | 64 | 84 |  |
| OCL | | | | | |
|  |  |  |  |  |  |
